# Prevention of Parastomal Hernia and the Prophylactic Mesh Debate: A Critical Appraisal of the Chimney Trial

**DOI:** 10.7759/cureus.110470

**Published:** 2026-06-08

**Authors:** Mohamed Alkashty, Ehab Kahka

**Affiliations:** 1 General Surgery, Wexham Park Hospital, Slough, GBR; 2 General Surgery, Cairo University Hospitals, Cairo, EGY; 3 General Surgery, Ain Shams University Hospital, Cairo, EGY

**Keywords:** chimney trial, colorectal surgery, end colostomy, parastomal hernia, prophylactic mesh

## Abstract

Parastomal hernia remains one of the most common long-term complications following permanent end colostomy, with significant implications for patient quality of life and healthcare resources. The recently published Chimney Trial reported a reduction in both radiological and clinically diagnosed parastomal hernia using a funnel-shaped prophylactic mesh during elective minimally invasive rectal cancer surgery. This editorial critically examines the trial's findings, strengths, limitations, and implications for clinical practice. While the results are encouraging, important questions remain regarding patient selection, mesh design, long-term safety, external validity, and clinically meaningful outcomes. Rather than closing the debate on prophylactic mesh, the Chimney Trial may redefine it by shifting attention towards the role of mesh configuration and patient-centered outcome measures.

## Editorial

Parastomal hernia is a common and clinically important complication after permanent end colostomy, with consequences ranging from appliance difficulty and discomfort to obstruction, impaired body image, loss of independence, and reoperation. Its prevention has therefore become a priority in colorectal and abdominal wall surgery. Yet, despite decades of technical innovation, the role of prophylactic mesh at the time of stoma formation remains contested.

The rationale for prophylactic mesh is compelling. Reinforcement at the trephine may reduce progressive fascial enlargement and prevent clinically significant herniation. Early randomised studies suggested benefit, and the European Hernia Society guidance supports the use of prophylactic synthetic non-absorbable mesh during elective permanent end colostomy construction, particularly in patients at high risk of parastomal hernia [1,2]. However, subsequent multicentre randomised trials, including STOMAMESH (Use of Prophylactic Mesh When Creating a Colostomy Does Not Prevent Parastomal Hernia) and GRECCAR 7 (End Colostomy With or Without Mesh to Prevent a Parastomal Hernia), challenged this assumption by showing no clear reduction in parastomal hernia with flat keyhole techniques [3-5].

Against this background, the Chimney Trial is important because it tests a different concept: a three-dimensional funnel-shaped intra-abdominal mesh designed to accommodate the bowel and reinforce the abdominal wall without relying on a flat keyhole configuration [6-8]. The three-year follow-up report shows a significant reduction in both radiological and clinical parastomal hernia, without an apparent increase in mesh-related complications [6]. This appraisal examines whether these findings are sufficient to change practice or should instead be used to refine the questions surgeons ask about mesh design, patient selection, endpoints, and long-term safety.

Overview of the chimney trial

The Chimney Trial was a randomised, single-blinded, multicentre study conducted in Finland and Sweden. Patients undergoing laparoscopic or robotic abdominoperineal excision or low Hartmann procedure for rectal adenocarcinoma with permanent end colostomy were randomised to receive a funnel-shaped polyvinylidene fluoride intra-abdominal mesh or no mesh.

At three years, 50 patients in the mesh group and 51 in the control group were available for clinical analysis, with computed tomography performed in 44 and 39 patients, respectively. CT-confirmed parastomal hernia occurred in 25 of 44 patients in the mesh group compared with 32 of 39 controls, an absolute difference of −25%. Clinically diagnosed parastomal hernia occurred in 10% of mesh patients compared with 39% of controls. Hernias were also smaller in the mesh group, with lower median hernia volume and smaller fascial defects. No significant difference was reported in stoma-related complications, small bowel obstruction, reoperation, or quality-of-life scores [6].

These findings are clinically attractive. However, a critical appraisal must distinguish statistical significance from clinical certainty.

Internal validity: a well-designed trial with important caveats

The trial has several major strengths. It was randomised, multicentre, and prospectively designed, using a standardised mesh and technique [6-8]. Radiological assessment was performed by blinded radiologists, reducing detection bias for the imaging endpoint. The intervention was clearly defined, and the trial addressed a focused clinical question in a population where permanent colostomy is common and parastomal hernia prevention is relevant.

However, the trial was terminated prematurely because the control group exceeded a prespecified clinical threshold for parastomal hernia at 12 months. This was ethically understandable but methodologically important. Trials stopped early for apparent benefit may overestimate treatment effects, particularly when event numbers are small, or follow-up is incomplete [9]. The Chimney findings are therefore persuasive but should not be interpreted as definitive.

Attrition is another concern. At three years, CT imaging was available for only 44 patients in the mesh group and 39 in the control group. Loss to follow-up is not merely a logistical issue; it may threaten internal validity if patients who are lost to follow-up differ systematically from those assessed [10]. In a cancer population, death, recurrence, frailty, and inability to attend imaging may all relate to competing risks and overall abdominal wall outcomes. The direction of bias is uncertain, but its presence cannot be dismissed.

Radiological success versus clinical reality

The most intellectually important finding is not simply that the mesh reduced parastomal hernia. It is that 57% of mesh patients still had CT-confirmed parastomal hernia at three years, while only 10% had clinically diagnosed hernia. This discrepancy is central to interpretation.

If the aim of prophylactic mesh is to prevent any radiological trephine abnormality, the intervention is only partially successful. If the aim is to prevent symptomatic, palpable, progressive, or surgically relevant parastomal hernia, the result is more impressive. The trial, therefore, challenges the field to define what outcome matters most. A radiological endpoint is objective and reproducible, but may overdiagnose clinically irrelevant disease. A clinical endpoint is more patient-centred but vulnerable to detection bias when patients and clinicians are not fully blinded [11].

This distinction matters because surgeons do not treat scans; they treat patients. The reduction in clinical parastomal hernia from 39% to 10% is compelling, but the absence of a significant quality-of-life difference tempers the claim that the intervention has already demonstrated patient-centred superiority. Future studies should prioritise symptomatic hernia, appliance problems, patient-reported burden, hernia progression, reoperation, and cost-effectiveness alongside radiological diagnosis.

Mesh design may be the missing variable

The Chimney Trial should not be read simply as “mesh works.” A more accurate interpretation is that mesh design may matter. Previous keyhole mesh trials, including STOMAMESH and GRECCAR 7, failed to demonstrate convincing benefit [3-5], whereas the Chimney Trial suggests that a three-dimensional, funnel-shaped intra-abdominal construct may reduce clinically relevant herniation [6].

This is an important conceptual shift. The question is no longer whether prophylactic mesh as a broad category is effective but whether specific geometry, position, fixation, material, and surgical context determine success. A flat keyhole mesh may fail because the trephine enlarges, the bowel acts as a dynamic piston, or the mesh aperture becomes the point of weakness. A funnel-shaped mesh attempts to address this by creating a three-dimensional reinforced channel around the bowel. This biological and mechanical rationale makes the Chimney result plausible [12-15].

Recent systematic reviews and network meta-analyses suggest that prophylactic mesh reduces the overall risk of parastomal hernia, but substantial heterogeneity remains across stoma types, mesh planes, definitions, and follow-up periods [16]. The Chimney Trial adds high-quality evidence, but it does not erase the inconsistency of the wider literature.

External validity: who is this trial really about?

The study population was carefully selected: elective rectal adenocarcinoma patients undergoing laparoscopic or robotic abdominoperineal excision or low Hartmann procedure, with emergency surgery, open conversion, active infection, T4b multivisceral resections, and several high-risk groups excluded. Mean BMI was approximately 26.5 kg/m², limiting direct applicability to obese populations.

This matters because many patients who develop parastomal hernia in routine practice are older, obese, frail, comorbid, or undergo emergency surgery. The trial therefore supports consideration of a funnel-shaped mesh in selected elective, minimally invasive rectal cancer cases, but it does not justify uncritical use in all permanent colostomies. General district hospitals may also face a learning curve, cost constraints, procurement issues, and variable access to surgeons experienced in intra-abdominal mesh placement.

Safety and the need for longer follow-up

The absence of increased complications at three years is reassuring. The trial reported no significant increase in stoma stricture, small bowel obstruction, reoperation, or other stoma-related complications. However, mesh-related complications may present late, and three years is relatively short for assessing erosion, chronic infection, fistulation, pain, or difficulty with reoperation. The planned five-year follow-up will be important, but even longer registry-based surveillance may be required before broad adoption.

The conflict-of-interest statement also deserves transparent consideration. The lead author reported personal fees from Medtronic and DynaMesh and grants from the European Hernia Society and Ella & Georg Ehrnrooth Foundation, while the funders were reported to have no role in study design, conduct, analysis, or publication[6]. This does not invalidate the findings, but device trials require scrutiny because technical enthusiasm and commercial interests can influence adoption before long-term evidence matures.

Implications for practice

The Chimney Trial should influence practice, but selectively. In a patient undergoing elective laparoscopic or robotic abdominoperineal excision or Hartmann procedure for rectal adenocarcinoma, particularly with reasonable life expectancy and acceptable contamination risk, prophylactic funnel-shaped mesh is now a serious option. It may reduce the incidence of clinically significant parastomal hernia and appears safe in the studied setting.

However, it should not yet be treated as a universal standard for all permanent colostomies. Evidence remains insufficient for emergency stomas, open procedures, severe obesity, active infection, inflammatory bowel disease, palliative surgery with limited survival, or non-specialist settings without appropriate training. The correct message is not “apply mesh to everyone,” but “consider the right mesh in the right patient, using the right technique, and measure the right outcome.”

Future directions

Future research should move beyond binary mesh-versus-no-mesh comparisons. The next generation of studies should compare mesh geometry, placement plane, fixation method, and patient selection. Trials should stratify by BMI, diabetes, chemotherapy, abdominal wall hernia history, sarcopenia, surgical approach, and stoma site. Outcomes should include symptomatic parastomal hernia, appliance difficulty, patient-reported quality of life, hernia progression, reoperation, cost-effectiveness, and long-term mesh complications.

Pragmatic implementation studies are also needed. A technique that works in expert Scandinavian centres may not reproduce identical results in lower-volume units without structured training. Registry-based follow-up could capture late complications and real-world effectiveness more efficiently than repeated small trials.

Conclusion

The Chimney Trial is one of the most important contemporary studies in parastomal hernia prevention. It provides credible evidence that funnel-shaped intra-abdominal mesh can reduce both radiological and clinically diagnosed parastomal hernia after elective minimally invasive rectal cancer surgery, without an early signal of harm. Yet the trial does not close the debate. Instead, it reframes it.

The question of the future is no longer simply whether prophylactic mesh should be used. It is whether specific mesh designs can prevent clinically meaningful parastomal hernia in carefully selected patients without introducing late harm, unnecessary cost, or radiological overdiagnosis. The Chimney Trial opens that door convincingly. It has not yet closed it.
